# Tranexamic acid to prevent operation in chronic subdural haematoma (TORCH): study protocol for a randomised placebo-controlled clinical trial

**DOI:** 10.1186/s13063-021-05907-0

**Published:** 2022-01-18

**Authors:** S. Immenga, R. Lodewijkx, Y. B. W. E. M. Roos, S. Middeldorp, C. B. L. M. Majoie, H. C. Willems, W. P. Vandertop, D. Verbaan

**Affiliations:** 1 Department of Neurosurgery, Amsterdam UMC, University of Amsterdam, Amsterdam, The Netherlands; 2 Department of Neurology, Amsterdam UMC, University of Amsterdam, Amsterdam, The Netherlands; 3 Department of Vascular Medicine, Radboud University Medical Center, Nijmegen, The Netherlands; 4 Department of Radiology, Amsterdam UMC, University of Amsterdam, Amsterdam, The Netherlands; 5 Department of Internal Medicine, Geriatrics Section, Amsterdam UMC, University of Amsterdam, Amsterdam, The Netherlands

**Keywords:** Activities of daily living, Antifibrinolytic agents, Neurosurgery, Haematoma, Subdural, Chronic, Conservative treatment, Tranexamic acid, Quality of life

## Abstract

**Background:**

Chronic subdural haematoma (cSDH) occurs mainly in the elderly. Surgical evacuation is effective, but in these old, often frail, patients with multi-comorbidity, surgery carries significant risks for future cognitive functioning and loss of independency. Therefore, a growing interest is noted for a non-surgical treatment with medication such as tranexamic acid (TXA). In five small retrospective series, this antifibrinolytic drug showed a beneficial effect on the spontaneous resolution of the haematoma, and with that, the necessity for surgery.

**Methods:**

For this randomised, placebo-controlled clinical multicentre trial, all cSDH patients, over 50 years old with mild symptoms (Glasgow Coma Score (GCS) ≥ 14, modified National Institutes of Health Stroke Scale (mNIHSS) ≤ 4), a midline shift of ≤ 10 mm and in whom a primary conservative treatment is chosen, are eligible for study participation. After informed consent, 140 patients will be randomised to receive either TXA 500 mg or placebo two times daily for 28 days. The primary outcome is the necessity for surgery within 12 weeks; secondary outcomes are cSDH volume, neurological impairment (mNIHSS), falling incidents, cognitive functioning (Montreal Cognitive Assessment (MOCA)), performance in activities of daily living (Barthel and Lawton score), functional outcome (modified Rankin Scale (mRS)), quality of life (Short Form Health Survey (SF-36) and EuroQol 5-Dimension Health Survey (EQ-5D)), mortality and the use of care and health-related costs (Medical Consumption Questionnaire (iMCQ) and Productivity Cost Questionnaire (iPCQ)) at 12 weeks and 6 months.

**Discussion:**

This phase III trial investigating the efficacy of TXA to prevent surgery for cSDH is the first in including patients using anticoagulants and mentally incompetent patients, since these comprise a significant part of the target population. Also, this study is one of the first to prospectively measure functional outcome and quality of life in cSDH patients. Final results of this study are expected in 2024.

**Trial registration:**

Dutch Trial Registry (Nederlands Trial Register) NL6584. Registered on 11 November 2017

ClinicalTrials.govNCT03582293. Registered on 11 July 2018

EU Clinical Trials Register 2017-004311-40. Registered on 29 March 2018

## Administrative information

Note: the numbers in curly brackets in this protocol refer to SPIRIT checklist item numbers. The order of the items has been modified to group similar items (see http://www.equator-network.org/reporting-guidelines/spirit-2013-statement-defining-standard-protocol-items-for-clinical-trials/).

**Table:** 

Title {1}	Tranexamic acid to prevent operation in chronic subdural hematoma (TORCH): study protocol for a randomized and placebo controlled trial
Trial registration {2a and 2b}.	Dutch Trial Registry (Nederlands Trial Register): NTR6758. Registration date: November 11, 2017Clinicaltrials.gov: NCT03582293. Registration date: July 11, 2018EU Clinical Trials Register: 2017-004311-40. Registration date: March 29, 2018
Protocol version {3}	Version: 1.3, date: February 19, 2019
Funding {4}	The trial is funded by a grant obtained from ZonMW, the Netherlands Organisation for Health Research and Development. ZonMW approved the study protocol. The study is registered under project number 848081003.
Author details {5a}	S. Immenga ^1^, MD (SI, corresponding author)s.immenga@amsterdamumc.nl R. Lodewijkx ^1^, BSc (RL) r.lodewijkx@amsterdamumc.nl Y.B.W.E.M. Roos ^2^, MD PhD (YR)y.b.roos@amsterdamumc.nl S. Middeldorp ^3^, MD PhD (SM) saskia.middeldorp@radboudumc.nl C.B.L.M. Majoie ^4^, MD PhD (CM)c.b.majoie@amsterdamumc.nl H.C. Willems ^5^, MD PhD (HW)h.c.willems@amsterdamumc.nl W.P. Vandertop ^1^, MD PhD (WV)wp.vandertop@amsterdamumc.nl D. Verbaan ^1^, PhD (DV)d.verbaan@amsterdamumc.nl ^1^ Department of Neurosurgery, Amsterdam UMC, University of Amsterdam, Amsterdam, The Netherlands ^2^ Department of Neurology, Amsterdam UMC, University of Amsterdam, Amsterdam, The Netherlands ^3^ Department of Vascular Medicine, Radboud University Medical Center, Nijmegen, The Netherlands ^4^ Department of Radiology, Amsterdam UMC, University of Amsterdam, Amsterdam, The Netherlands ^5^ Department of Internal Medicine, Geriatrics Section, , Amsterdam UMC, University of Amsterdam, Amsterdam, The Netherlands
Name and contact information for the trial sponsor {5b}	Amsterdam University Medical Centers, location Academic Medical Center, PO Box 22660, 1100 DD, Amsterdam, the Netherlands.
Role of sponsor {5c}	Since this is an investigator-initiated study, the sponsor has had no role in the setup of the study design. The sponsor only requires that the execution of the study is performed according to the ICH guidelines for Good Clinical Practice. The funder was involved in setting up the study design and approved the study protocol as presented in this article. The funder has no role in the data collection, management, analysis and interpretation, other than that they require that the study data becomes publically available after completion and publication of the study.

## Introduction

### Background and rationale {6a}

Chronic subdural haematoma (cSDH) is a frequently occurring neurological disease in elderly [1, 2]. It consists of an extracerebral encapsulated collection of mostly liquefied old haematoma, located between the dura and arachnoid. The original small, and often asymptomatic, haemorrhage is caused by rupture of a bridging vein usually after, often minor, head trauma. Due to generalised cerebral atrophy, increased venous fragility [3] and more frequent use of anticoagulation therapy, older people are more at risk of developing a cSDH.

During several weeks, the original haematoma is encapsulated by a membrane consisting of weak neocapillaries from where recurrent small bleedings occur. The pathophysiological mechanism is thought to be a problem in the local haemostasis due to fibrin degradation products of the original haematoma that inhibit further haemostasis in the subdural space [4]. Together with an osmotic draw of water, owing to its high protein content, this results in the growth of the haematoma. Therefore, it usually takes several weeks for the cSDH to grow and become symptomatic due to compression on the brain parenchyma.

The current incidence of cSDH is estimated to be 15/100,000 per year [5]. The number of patients with cSDH is expected to increase considerably in the near future due to the continuing growth of the elderly population and the increase in the use of anticoagulation and antiplatelet aggregation therapy [6]. By 2030, the incidence is expected to rise up to 20/100,000 per year [5], thus making cSDH the most common intracranial neurosurgical condition in adults.

#### Current treatment

Currently, accepted treatment options are conservative and surgical. Conservative treatment consists of a wait-and-scan policy in which the patient is regularly monitored for neurological deterioration and growth of the haematoma on follow-up imaging. Anticoagulation and antiplatelet aggregation therapy is discontinued in low-risk patients, based on individual risk-benefit assessment and the discretion of the treating physician. In the Netherlands, about 50% of all cSDH patients is treated conservatively [7]. Spontaneous resolution of cSDH with a conservative treatment occurs in approximately 2.5% of patients however and is therefore relatively rare [8]. If the conservative treatment fails, surgical treatment has to be considered. In our own data (unpublished), 75% of the cSDH patients require surgery after a failed conservative treatment.

Surgical evacuation of the haematoma is currently the designated therapy in patients with severe symptoms or if the primary conservative treatment fails. Surgery usually consists of drainage of the liquefied haematoma through a burr hole craniostomy under general anaesthesia. This treatment is effective, but is also associated with life-threatening risks in these old, often frail, patients with multi-comorbidity. Postoperative complications such as a delirium and pneumonia can lead to a deterioration in cognitive functioning, loss of independency and even death. In a large series of 1205 patients, symptomatic recurrence after surgery was 9%, mortality 2% and unfavourable functional outcome 22% [9].

#### Tranexamic acid

As hyperfibrinolysis is thought to play a role in the liquefaction and enlargement of cSDH, pharmaceutical options to block fibrinolysis have been explored in an effort to eliminate the necessity for surgery [10–16]. The use of tranexamic acid (TXA), an antifibrinolytic drug, has so far been reported in five small series. In the first retrospective series, a total of 21 patients were treated with TXA, of whom three after primary burr hole surgery. In none of these 21 patients, additional surgery was necessary [13]. The second, a prospective pilot study in 14 patients, showed a 41% reduction of cSDH after surgery and an additional 91% residual volume reduction on CT after 90 days during oral TXA treatment of 650 mg per day for a mean (*SD*) duration of 90 (27) days, without recurrence, re-expansion or any complicating venous thromboembolisms [14]. The third study, a case report series of three patients treated with 650 mg TXA per day for 1 month after surgery for cSDH, showed no recurrences and thromboembolic complications [12]. The fourth, a case report of one patient successfully treated primarily with TXA, was recently published [15], and finally, in an Asian article, the authors conclude that administration of Gorei-San, a Japanese herbal Kampo medicine, combined with TXA has the potential to prevent recurrences of cSDH [16].

With these limited, however promising, data, no definitive conclusion can be made regarding the role of TXA in the treatment of cSDH. Therefore, a prospective study evaluating the efficacy and safety of TXA is needed. Currently, two prospective trials (the TRACS study [17] and the TRACE study [18]) are running in an effort to answer this question. The first (TRACS), a phase IIb trial with the aim to provide preliminary data required to plan a larger phase III trial, excludes patients using anticoagulants [17]. These patients comprise a significant portion of the cSDH patient population, and therefore, the results of the TRACS study will be difficult to extrapolate to the future care of all cSDH patients. The second (TRACE) is a randomised, observer-blinded trial, investigating the value of treating cSDH patients with TXA after surgery [18]. In our trial, we plan to include only patients in whom the primary treatment is conservative. Both trials are set up with a primary radiological outcome parameter and therefore potentially provide insufficient clinically relevant information.

#### Risks

Opposed to what is commonly thought, TXA has no known prothrombotic effects. It is an antifibrinolytic drug that inhibits the action of plasmin. Several studies with TXA show no increase in fatal or non-fatal vascular occlusive events (1.7% TXA versus 2.0% placebo; *RR* (95% *CI*) 0.84 (0.68–1.0)) [19] or death or thrombotic complications (*RR* (95% *CI*) 0.92 (0.81–1.05)) [20]. In a Swedish case-control study of 1955 women, no increased risk of venous thromboembolism for women using TXA for menorrhagia was found (*OR* 0.55 (0.31–0.97)) [21]. Also, in another study evaluating the effect of high-dose (1 g, 3 times daily) TXA on epistaxis in 135 patients with hereditary haemorrhagic telangiectasia, no thromboembolic events were seen after 3 months (incidence 0%, 95% *CI* 0.0–2.8%) [22]. A recent systematic review on the use of TXA in non-surgical patients showed a reduced all-cause mortality without increased risk of venous or arterial thrombotic complications (22 studies representing 49,538 patients) [23].

## Objectives {7}

Our phase III trial aims to investigate the efficacy of TXA as an addition to a primary conservative treatment of cSDH, in an effort to prevent surgery for cSDH. Since surgical treatment is associated with significant morbidity [9], we assume that preventing surgery also prevents deterioration of patients’ cognitive functioning, loss of independency and even death due to postoperative complications. If this study shows that TXA is an effective treatment, steps will be taken to register cSDH as a new indication for TXA. Together with the registration, the use of TXA can be incorporated in the clinical guideline for the treatment of cSDH.

## Trial design {8}

This multicentre study is designed as a double-blind, placebo-controlled, randomised, clinical superiority trial. Randomisation will be performed with a 1:1 allocation ratio.

## Methods: participants, interventions and outcomes

### Study setting {9}

**Fig. 1:**
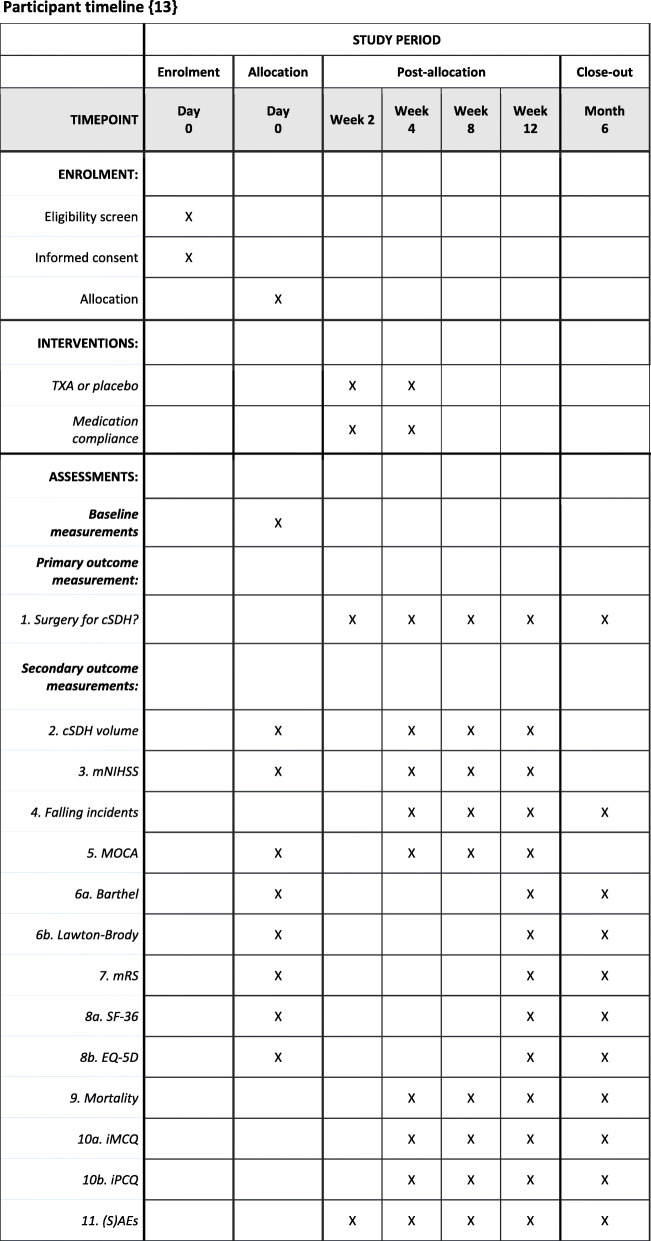
Participant timeline

In the participating centres, all patients who are diagnosed with a cSDH are tested for eligibility for study participation. The treatment strategy, either primarily surgical or primarily conservative, will be decided on the basis of clinical and radiological parameters. Screening for inclusion will be performed when a conservative treatment strategy is selected. After gaining informed consent, the patient is randomised to either the treatment or the placebo group. Treatment starts within 24 h for a total of 28 days. Study participants are being monitored according to the follow-up schedule (Fig. 1).

### Eligibility criteria {10}

Inclusion criteria are (1) age ≥ 50 years, (2) on CT-confirmed cSDH and (3) primary conservative treatment, based on clinical symptoms: Glasgow Coma Scale (GCS) score ≥ 14, modified National Institutes of Health Stroke Scale score ≤ 4 and a stable neurological deficit (no new, or progression of, symptoms until assessment for study inclusion).

Exclusion criteria are (1) primary surgical treatment, based on one or more of the following symptoms or parameters: midline shift > 10 mm (judged by a second independent neurosurgeon if > 5 mm) and imminent death within 24 h; (2) structural causes for subdural haemorrhage, e.g. arachnoid cysts, cortical vascular malformations and a history of cranial surgery < 1 year; (3) aneurysmal subarachnoid haemorrhage; (4) active treatment for deep vein thrombosis, pulmonary embolism or cerebral thrombosis (secondary prophylaxis is not considered to be active treatment); (5) active intravascular clotting or disseminated intravascular coagulation; (6) known hypersensitivity or allergy to TXA or to any of the ingredients; (7) blood coagulation disorder; (8) severe renal impairment; (9) anaemia; (10) history of convulsions; (11) inability to safely swallow oral medication; and (12) inability to obtain informed consent from patient or legal representative (in case of depressed level of consciousness).

### Who will take informed consent? {26a}

Informed consent will be obtained by the patients’ treating physician, together with one of the researchers authorised for this task. An informed consent form will be signed by one of the researchers and the trial participant, or authorised surrogate if the trial participant has a decreased level of consciousness. In the latter case, informed consent will be obtained in the second instance if the trial participant becomes mentally competent during the study follow-up.

### Additional consent provisions for collection and use of participant data and biological specimens {26b}

Together with informed consent for participation in the study, additional consent is asked for reuse of the pseudonymised collected data for future research questions. For this study, no biological specimens will be collected.

## Interventions

### Explanation for the choice of comparators {6b}

Study treatment consists of the oral administration of a capsule with either tranexamic acid or a placebo substance, both in addition to standard care. A placebo is used as a comparator to exclude the bias where the decision to perform a surgical treatment is influenced by the use of tranexamic acid.

### Intervention description {11a}

The intervention consists of the oral administration of a capsule with either 500 mg tranexamic acid or a placebo substance twice daily for a period of 4 weeks (28 days), both in addition to standard care. If surgery for the cSDH is necessary, administration of the study medication is stopped directly after surgery.

Standard care consists of cessation of anticoagulant of antiplatelet aggregation depending on an individual risk assessment, closely monitoring for new neurological symptoms during hospital admission and/or during follow-up in the outpatient clinic, radiological monitoring with repeat CT scans every 4 weeks and admission to a rehabilitation centre if necessary. Follow-up takes several months, depending on the rate of improvement of symptoms. If new neurological symptoms arise or if existing neurological symptoms do not improve based on a progressive or non-resolving haematoma, surgical treatment is reconsidered.

### Criteria for discontinuing or modifying allocated interventions {11b}

The study intervention cannot be modified during study participation. It is not necessary to adjust the dose for renal function as the used dosage is safe up to a creatinine level of 500 μmol/L, as specified in the exclusion criteria. Also, interpretation of the study results is easier when the treatment is uniform. Therefore, study participants are not allowed to use tranexamic acid during study participation.

Crossover to the other study arm is not possible: study treatment stops if a surgical treatment for the cSDH is necessary during follow-up, if cessation of the treatment is necessary due to a suspected serious adverse reaction or if the trial participant decides to stop study participation.

### Strategies to improve adherence to interventions {11c}

Study treatment takes place during the first 4 weeks after inclusion. Follow-up visits take place at 2, 4, 8 and 12 weeks. At inclusion, the participant receives instructions about taking the medication including timing, storage, the importance of taking the capsules whole and what to do in the event of a missed dose. Also, the importance of adherence to the study protocol is discussed. Any leftover study medication is counted during the follow-up visit at 4 weeks. If protocol adherence has been good, the participant should return an empty bottle. Besides these oral instructions, study participants also receive these instructions on paper.

During follow-up visits at 2 and 4 weeks, treatment adherence is monitored by asking at what times the capsules are ingested and whether the participant experiences any side effects. Study participants are asked to contact one of the researchers with any questions about the study medication during the treatment period. The drug monitoring is performed by one of the researchers or by the research nurse who will also perform the follow-up outcome measurements. If necessary, the treating physician is consulted.

At 4 weeks, the participant will return the study medication bottle after which any leftover medication is counted. The number of unused capsules and the reason for possible non-compliance (if one or more capsules are returned) is registered in the designated case report form.

### Relevant concomitant care permitted or prohibited during the trial {11d}

Besides treatment with TXA, any other medical treatments are permitted during study participation.

### Provisions for post-trial care {30}

Parallel to study participation, all study participants receive standard care. This includes extended follow-up after study participation has ended, if deemed necessary by the treating physician. All study participants can claim reimbursement from the study insurance, if they have suffered harm from trial participation.

### Outcomes {12}

The primary endpoint is the number of patients requiring surgery for chronic subdural haematoma within 12 weeks after the start of treatment with the study medication.

Secondary endpoints are (1) number of patients requiring surgery within 6 months; (2) cSDH volume measured on non-contrast head CT (NCCT) with Brainlab Cranial Planning [24] at 4, 8 and 12 weeks; (3) neurological impairment (mNIHSS [25] score) at 4, 8 and 12 weeks; (4) number of falling incidents at 12 weeks; (5) cognitive functioning (Montreal Cognitive Assessment, MOCA score [26]) at 4, 8 and 12 weeks; (6) performance in activities of daily living (Barthel [27] and Lawton-Brody [28] score) at 12 weeks and 6 months; (7) functional outcome (modified Rankin Scale, mRS [29] score) at 12 weeks and 6 months; (8) quality of life (Short Form Health Survey, SF-36 [30] and five-dimensional EuroQol, EQ-5D-3L [31]) at 12 weeks and 6 months; (9) mortality at 12 weeks and 6 months; and (10) care and health-related costs (Medical Consumption Questionnaire, iMCQ [32] and Productivity Cost Questionnaire, iPCQ [32]) at 12 weeks.

### Participant timeline {13}

The participant timeline is shown in Fig. 1.

### Sample size {14}

In the Netherlands, approximately 50% of patients with cSDH is primarily treated conservatively [7]. Of these, 75% still need surgery (own data). Since oral TXA is an innovative treatment in these patients, little data is available concerning its efficacy. Until today, only five small studies, concerning a total of 39 patients treated with TXA, have been published [12–16]: none of these patients required surgery after the start of treatment. As this 100% reduction of surgery may well be an over-estimation of the true effect, a conservative estimate of 33% relative risk reduction was used in the sample size calculation (from 75% surgery in the placebo group to 50% surgery in the TXA group). A Fisher’s exact test with a 0.05 two-sided significance level will have 80% power to detect the difference between a control (placebo) group proportion of 0.75 and a treatment (TXA) group proportion of 0.50 when the sample size in each group is 64 (128 patients in total).

We consider the quality of life (QoL), measured with the SF-36, as an important secondary functional outcome indicator. With a sample size of 64 patients per treatment arm, we are also able to detect a Cohen’s *d* effect size (difference between the mean SF-36 scores of the control group and treatment group divided by the pooled SD) of 0.50. Although an effect size of *d* = 0.50 can be defined as ‘moderate’ [33], such a difference in mean QoL scores may be clinically important. Anticipating an attrition rate of about 8%, we will include 70 (64/0.92) patients in each group (140 patients in total).

### Recruitment {15}

All physicians in participating centres treating cSDH patients are made aware of the study, so that study inclusion is considered in every cSDH patient. Treating physicians of non-participating medical centres are made aware of this study with recurrent oral presentations, in an effort to stimulate referrals to a participating centre, so that study inclusion can be considered. Also, the study has been promoted with an article in the *Dutch Journal of Medicine* [34] and with a website with all relevant information for caretakers and patients [35].

## Assignment of interventions: allocation

### Sequence generation {16a}, concealment mechanism {16b} and implementation {16c}

Before the start of the study, a randomisation list was made by a statistician using an online randomisation module (TENALEA Clinical Trial Data Management System) and random blocks of sizes 2, 4 and 6 stratified for anticoagulant and/or antiplatelet use (yes/no). A 1:1 ratio for either TXA or placebo was used. During inclusion, new participants are assigned to the sequential randomisation number from the randomisation list with the same online randomisation module. The pharmacy provides the participant with the study medication based on the randomisation number. The pharmacy is the only holder of the randomisation list. Concealment of treatment allocation is ensured, and patients, treating physicians and endpoint assessors are unaware of the treatment assignment.

## Assignment of interventions: blinding

### Who will be blinded {17a}

Trial participants, treating physicians and outcome assessors are blinded for the treatment assignment. This is ensured by a randomisation list which is held by the pharmacy only. This list contains the randomisation numbers and the assigned treatment group. During enrollment, the participant is assigned a randomisation number on which the pharmacy hands out coded study medication.

The study medication consists of capsules which look the same in both treatment groups. The container holds a label with only the randomisation number and name of the participant.

### Procedure for unblinding if needed {17b}

The pharmacy will hold the unblinding codes. In case a SUSAR is suspected, the investigator/attending physician will email/fax an Unblinding Request Form (URF) to the Principal Investigator or delegated person and make every effort to contact the Principal Investigator to discuss options. In case unblinding is deemed necessary, the Principal Investigator or delegated person will send the URF to the pharmacy who will reveal the treatment assignment for the individual subject to the local investigator by telephone and confirmed in writing. The local investigator will document the unblinding on the Unblinding Form (UF) and store it in the local investigator study file (ISF). The date, time and reason for unblinding will also be recorded in the source documents and in the subject’s CRF.

If the blind is broken, the individual subject must be discontinued from the investigational medicinal product as soon as possible, when not already done so. The subject should be strongly encouraged to perform an end of study assessment and be under medical supervision until symptoms cease or the condition becomes stable.

An independent physician, authorised for this task, will report the SUSAR to ToetsingOnline in order to maintain the blind for the Principal Investigator and other research team members. The Principal Investigator or delegated person will file the URF in the Trial Master File.

## Data collection and management

### Plans for assessment and collection of outcomes {18a}

Research data is collected in an electronic CRF using Castor EDC (http://www.castoredc.com). Paper questionnaires are used for the SF-36, EQ-5D, iMCQ and iPCQ outcome measurements, which are filled in at home by the study participants themselves. These completed questionnaires are digitalised to Castor EDC by the investigators. During the outpatient clinic visit, the MOCA is assessed by the investigators using the official MOCA paper sheet, which is afterwards digitalised to Castor EDC by the investigators. The results of the mNIHSS, mRS, Barthel and Lawton-Brody are added to Castor EDC using direct entry during the outpatient clinic visit. Baseline patient characteristics, if a surgery for cSDH has been performed (primary outcome measurement), the number of falling incidents and mortality are registered in the medical records and added to Castor EDC after the outpatient clinic visit. Radiological data is also stored in the medical records; volume measurements are performed after the outpatient clinic visit and are then added to Castor EDC.

All outcome assessors are GCP licensed and are trained by the Principal Investigator in performing outcome measurements. The GCP certificates and training logs are stored in the Trial Master File. All electronic CRF entries are verified by checking the source data (paper questionnaires and medical records) which is done by the Principal Investigator or a delegated person.

### Plans to promote participant retention and complete follow-up {18b}

Study follow-up is performed at 4, 8 and 12 weeks with an outpatient clinic visit and CT scan. During these visits, all outcome measurements are performed by one of the researchers. To maximise participant retention, this study follow-up is parallel to the regular follow-up in the outpatient clinic by the treating physician (standard treatment). Also, we incorporate an extra telephonic interview after 2 weeks to monitor drug adherence and adverse events. Study treatment is limited to a maximum of 28 days and to a maximum of 56 capsules, regardless of whether a deviation from the study protocol occurs. In case of a protocol violation, study follow-up will remain unchanged.

### Data management {19}

Data management is described in the data management plan (DMP) which is available online [36].

### Confidentiality {27}

During the study, research data is pseudonymously collected in the electronic CRF (Castor EDC [37]). In every participating centre, a local key table is safely stored. This key table connects the research data to the patient data. The pseudonymous research data will only be shared after a data transfer agreement is signed. Research data is stored at the long-term storage facilities of the Academic Medical Center (Amsterdam, the Netherlands) for 15 years after completion of the study.

### Plans for collection, laboratory evaluation and storage of biological specimens for genetic or molecular analysis in this trial/future use {33}

Not applicable as no biological specimens are collected as part of this trial.

## Statistical methods

### Statistical methods for primary and secondary outcomes {20a}

Statistical analyses will be based on the intention-to-treat principle. Baseline assessments and outcome parameters will be summarised using simple descriptive statistics. Continuous, normally distributed variables will be expressed as means and standard deviations; continuous, non-normally distributed and ordinal variables as medians (25th–75th percentiles), and categorical variables as counts and percentages. Normality of data will be explored by a normal Q-Q plot and tested by the Shapiro-Wilk test. Where necessary we will use multiple imputations for handling missing data. In all analyses, statistical uncertainty will be expressed in two-sided 95% confidence intervals. A two-sided *p* value less than 0.05 is considered statistically significant. We will not correct for multiple testing.

The difference in the proportion of patients requiring surgery for cSDH within 12 weeks after the start of treatment will be analysed using Fisher’s exact test. In addition, logistic regression will be performed including treatment groups, stratification variables and baseline variables (if large differences exist between treatment groups) as independent variables. The effect size will be expressed in an adjusted odds ratio.

Differences in volume reduction of cSDH, neurological impairment (mNIHSS) and cognitive function (MOCA) between the treatment groups and overall time points will be analysed using a linear mixed model with treatment group membership as a fixed-effect and an appropriate random-effect structure. The number of falling incidents and mortality rate during the 12 weeks (and 6 months) follow-up will be analysed using Fisher’s exact test. ADL scores (Barthel Index and Lawton-Brody scale) and functional outcome score (mRS) at 12 weeks (and 6 months) will be compared with the two-sample *t*-test or Mann-Whitney test, where appropriate. Differences in the mean changes in the level of quality of life (SF-36) from baseline to 12 weeks (main secondary outcome) (and 6 months) will be analysed using the two-sample *t*-test. In addition, we will analyse these treatment effects by performing multivariable linear regression with treatment groups, the baseline values and the stratification variables as the independent variables.

For the cost-effectiveness analysis, the proportion of patients requiring surgery for cSDH within 12 weeks and 6 months after the start of treatment is the effect measure. The incremental cost-effectiveness ratio will be expressed as the costs per case of surgery avoided, as well as a cost-to-benefit ratio, where downstream costs associated with surgery and subsequent healthcare use until 12 weeks and 6 months are estimated. In addition, a cost-utility analysis (CUA) will evaluate cost differences in relation to differences in quality-adjusted life-years (QALYs). This CUA will estimate costs per QALY, to allow comparison with other health-related interventions or programmes. With a study horizon of 12 weeks and 6 months, no discounting will be applied.

We will differentiate between direct medical costs (surgical procedures, CT scans, pharmacological therapy, hospital stay, outpatient care, admissions to nursing home and other primary and paramedical health care following discharge), direct non-medical costs (travel to and from healthcare providers) and indirect costs (lost productivity due to absence from paid work). Healthcare utilisation during the index hospitalisation will be documented in the clinical report form. Healthcare and other resource use following discharge will be collected with the iMTA Medical Consumption questionnaire and the Productivity Costs Questionnaire [32] at 4, 8 and 12 weeks and 6 months. Unit costs for healthcare use will be estimated according to the Dutch guideline for economic evaluation research [38]. Medication costs will be valued by market prices [39]. Health-related QoL will be collected at 12 weeks and 6 months with the EQ-5D. Utility values for EQ-5D scores will be based on Dutch estimates [40]. Utility scores will be uniformly interpolated, assuming a constant health state between subsequent assessments.

Cost-effectiveness will be evaluated by calculating the incremental cost-effectiveness ratios (Δcosts/Δeffects). Robustness of the results for uncertainty in parameter estimates and assumptions will be evaluated in sensitivity analyses, including the UK valuation of health states.

### Interim analyses {21b}

A Data and Safety Monitoring Board (DSMB) will perform an interim analysis to monitor safety data and sample size assumptions when follow-up is completed of the first 35 participants, 60 participants and 105 participants. In this analysis, unblinded data are assessed and the DSMB can advise to adjust the conduct, design or sample size or to terminate the study.

### Methods for additional analyses (e.g. subgroup analyses) {20b}

Additional analyses are described in the yet to be published statistical analysis plan (SAP).

### Methods in analysis to handle protocol non-adherence and any statistical methods to handle missing data {20c}

Methods in analysis to handle protocol non-adherence and missing data are described in the yet to be published statistical analysis plan (SAP).

### Plans to give access to the full protocol, participant-level data and statistical code {31c}

The datasets analysed during the current study are available from the corresponding author on reasonable request.

## Oversight and monitoring

### Composition of the coordinating centre and trial steering committee {5d}

The trial steering committee consists of the authors of the study protocol. SI, WV and DV developed the study protocol with input from YR, SM, CM and HW. RL is involved in performing the outcome measurements. SI, WV and DV are responsible for the data collection and management, SAE reporting and the coordination of participating centres. A DSMB is installed to oversee the safety and feasibility of the trial.

### Composition of the data monitoring committee, its role and reporting structure {21a}

The DSMB consists of a statistician/methodologist and two clinicians. All members are independent of the study. Its role is the monitoring of participant safety, the planned sample size assumptions, the efficacy and the overall conduct of the study. Subsequent meetings will be held at 25%, 43% and 75% of trial completion with interim analyses. The DSMB reports directly to the Principal Investigator. Further details can be found in the DSMB charter.

### Adverse event reporting and harms {22}

#### (Serious) adverse events

Adverse events (AE) are defined as any undesirable experience occurring to a subject during the study, whether or not considered related to the investigational product. A serious adverse event (SAE) is any untoward medical occurrence or effect that results in death, is life threatening (at the time of the event), requires hospitalisation or prolongation of existing inpatients’ hospitalisation, results in persistent or significant disability or incapacity, is a congenital anomaly or birth defect or is any other important medical event that did not result in any of the outcomes listed above due to medical or surgical intervention but could have been based upon appropriate judgement by the investigator. An elective hospital admission will not be considered as a serious adverse event.

#### Suspected unexpected serious adverse reactions (SUSAR)

Suspected unexpected serious adverse reactions (SUSAR) are all untoward and unintended responses to an investigational product related to any dose administered. Unexpected adverse reactions are SUSARs if the following three conditions are met: the event must be serious; there must be a certain degree of probability that the event is a harmful and an undesirable reaction to the medicinal product under investigation, regardless of the administered dose; and the adverse reaction must be unexpected, that is to say, the nature and severity of the adverse reaction are not in agreement with the product information as recorded in the Summary of Product Characteristics (SPC).

#### Reporting

Since the largest part of the study period takes place at home, AEs will not directly be observed by the investigator or his staff. Getting knowledge of AEs depends on spontaneous reporting by the subject or other treating physicians to the investigator or his staff. The reported AE will be documented in the patients’ medical file, triaged for being a possible SAE and, if so, handled as such. (S)AEs will be reported during the 12-week study period. If the participant opts in for the optional 6-month telephonic outcome measurement, (S)AEs will be reported during the additional study period between 12 weeks and 6 months after inclusion as well. Each AE will be reported in the CRF. (S)AEs should also be reported to the coordinating investigator/sponsor within 24 h.

The investigator will report all SAEs to the sponsor without undue delay after obtaining knowledge of the events, except for the following:

Hospital admission for the treatment of diseases that can be attributed to being an elderly patient, such as delirium, infections, constipation and an exacerbation of a pre-existent disease (excluding cSDH). These SAEs will be reported in a twice-yearly line listing until the follow-up of the last patient is completed

Hospital admission because of the necessity for surgical treatment of the cSDH. This SAE will be reported in a twice-yearly line listing, since this is the primary endpoint of our study

Except for the abovementioned, the sponsor will report the SAEs through the Web portal ToetsingOnline to the accredited METC that approved the protocol, within 7 days of first knowledge for SAEs that result in death or are life threatening followed by a period of maximum of 8 days to complete the initial preliminary report. All other SAEs will be reported within a period of maximum 15 days after the sponsor has first knowledge of the serious adverse events.

All AEs will be followed until they have abated, or until a stable situation has been reached. Depending on the event, follow-up may require additional tests or medical procedures as indicated, and/or referral to the general physician or a medical specialist.

SUSARs can only be determined by a physician. Therefore, if a trial nurse suspects an SAE to be a SUSAR, the adverse event is presented to the investigator. The investigator judges whether the event must be assessed as a SUSAR. The sponsor will report expeditiously the following SUSARs through the Web portal ToetsingOnline to the METC: SUSARs that have arisen in the clinical trial that was assessed by the METC and SUSARs that have arisen in other clinical trials of the same sponsor and with the same medicinal product and that could have consequences for the safety of the subjects involved in the clinical trial that was assessed by the METC.

The remaining SUSARs are recorded in an overview list (line-listing) that will be submitted once every half year to the METC. This line-listing provides an overview of all SUSARs from the study medicine, accompanied by a brief report highlighting the main points of concern. The expedited reporting will occur not later than 15 days after the sponsor has first knowledge of the adverse reactions. For fatal or life-threatening cases, the term will be maximal 7 days for a preliminary report with another 8 days for completion of the report.

### Frequency and plans for auditing trial conduct {23}

Academic Medical Center’s Clinical Research Unit (CRU) will provide independent monitoring. An independent monitor will monitor the study data according to Good Clinical Practice (GCP). In a selection of patients, informed consent will be checked. Additionally, there will be an onsite monitoring source data verification. The intensity for this verification is related to the risk analysis of the trial. Details will be described in a study-specific monitor plan. Monitoring will be performed after every third, 10th and 25th inclusion in a participating centre and after a total of 35, 60 and 105 inclusions. Close-out monitoring will be after the last visit of the last participant at all participating centres.

### Plans for communicating important protocol amendments to relevant parties (e.g. trial participants, ethical committees) {25}

A ‘substantial amendment’ is defined as an amendment to the terms of the METC application, or to the protocol or any other supporting documentation, that is likely to affect to a significant degree: the safety or physical or mental integrity of the subjects of the trial, the scientific value of the trial, the conduct or management of the trial or the quality or safety of any intervention used in the trial.

All substantial amendments will be notified to the METC and to the competent authority. Non-substantial amendments will not be notified to the accredited METC and the competent authority, but will be recorded and filed by the sponsor.

## Dissemination plans {31a}

The study will be registered in an international trial registry (http://www.clinicaltrials.gov). After completion of the study, the authors aim to publish the results in high-impact peer-reviewed journals and present the results in the usual international fora of relevant specialist societies, regardless of either positive or negative results. Authorship will be granted using the Vancouver definitions and depending on personal involvement. The first, second and last author names will be decided by the Principal Investigator and project leader. Besides the first, second and last authors, the steering group members and additional names are mentioned in alphabetical order. Participating centres including ≥ five patients will be entitled to one name in the author list. After the author list, the following sentence will be added: “on behalf of the TORCH-trial group” and a reference to an appendix with all sites, site investigators and number of patients enrolled.

In addition, if this study shows that TXA significantly prevents surgery for cSDH and, with that, improves the quality of life in elderly patients, steps will be taken to register cSDH as a new indication for TXA. Together with the registration, the use of TXA can be incorporated in the clinical guideline for the treatment of cSDH.

## Discussion

Optimal treatment for chronic subdural haematoma remains a matter of debate. Currently, the only effective treatment is surgical evacuation of the haematoma. In these old, often frail patients however, surgery comes with significant morbidity and mortality [9]. The quest for a non-surgical treatment has not yet resulted in an effective therapy. First reports on the use of TXA seem promising however [10–16]. This randomised controlled trial is meant to determine the efficacy of TXA as an addition to a primary conservative treatment of cSDH, in an effort to prevent surgery for cSDH.

Our study has several strengths. First, it tests the efficacy of TXA versus placebo during a conservative treatment of cSDH in an effort to prevent progression of the haematoma and with that, to prevent the necessity of a surgical treatment. Since progression of the haematoma is expected in the first weeks after diagnosis, study treatment is carried out during this period. Second, our primary endpoint is a clinical parameter instead of a radiological, in contrast to the two other currently running trials. This is important, because failure of the conservative treatment and the decision to perform an operation is based on clinical signs and symptoms and not so much on radiological parameters. Because we do think that radiological follow-up is important in the conservative treatment, we added it as a secondary outcome parameter. Third, we also added outcome parameters that measure cognitive function, functional outcome, quality of life and healthcare-related costs. All of these have only sparsely been reported in current literature. Fourth, we include mentally incompetent patients and patients using anticoagulants and antiplatelets which comprise a significant part of the target population. If we would not include them, it would be difficult to extrapolate the results of this study to the total group of patients with cSDH. Fifth, this study protocol does not interfere with our regular conservative treatment in which we monitor cSDH patients regularly and switch to a surgical if the symptoms worsen or do not recover. Finally, TXA is a safe drug with minimal side effects: most importantly, there is no evidence of an increased risk of thromboembolic complications when using TXA.

There are also some limitations of our protocol. First, a dosage of 1000 mg/day is given with the rationale that Dutch people on average are taller, and thus weigh more, than a previous cohort study in Japanese patients on 750 mg/day. It is unclear whether this dose is high enough. The dosage does however correspond to that given in the treatment of other diseases [41–43]. In addition, it is not known whether the length of treatment is adequate enough, and therefore, structured follow-up measurements at 4, 8 and 12 weeks will be done to evaluate the evolution of clinical symptoms and the subdural effusions, followed by a telephone interview at 6 months after inclusion to assess clinical status.

In conclusion, we have developed a protocol for a double-blind, placebo-controlled, multicentre and randomised clinical trial, evaluating the efficacy of TXA as an addition to a primary conservative treatment of cSDH, in an effort to prevent surgery for cSDH. In contrast to two currently running prospective trials, we have a clinical, instead of a radiological, primary endpoint. Therefore, this study should provide an answer whether surgery can be prevented and with that functional outcome improved, when treating cSDH patients with TXA.

## Trial status

Protocol version: 1.3

Protocol date: February 19, 2019

Start recruitment: June 19, 2018

End recruitment (approximate): March 2024

## Data Availability

The datasets analysed during the current study are available from the corresponding author on reasonable request.

## Abbreviations

AE: Adverse event
CRF: Case record form
CRU: Clinical Research Unit of the Academic Medical Center Amsterdam
cSDH: Chronic subdural haematoma
CT: Computed tomography
CUA: Cost-utility analysis
DMP: Data management plan
DSMB: Data safety and monitoring board
EQ-5D: EuroQol 5-dimension health survey
GCP: Good clinical practice
GCS: Glasgow Coma Scale
iMCQ: iMTA Medical Consumption Questionnaire
iPCQ: iMTA Productivity Cost Questionnaire
ISF: Investigator study file
MREC: Medical Research Ethics Committee
mNIHSS: Modified National Institutes of Health Stroke Scale
MOCA: Montreal cognitive assessment
mRS: Modified rankin scale
NCCT: Non-contrast head CT
RCT: Randomised controlled trial
QALY: Quality-adjusted life-years
QoL: Quality of life
SAE: Serious adverse event
SAP: Statistical analysis plan
SF-36: 36-Item short form health survey
SPC: Summary of product characteristics
SUSAR: Suspected unexpected serious adverse reaction
TMF: Trial master file
TXA: Tranexamic acid
UF: Unblinding form
URF: Unblinding request form
